# Increased Abundance of *Clostridium* and *Fusobacterium* in Gastric Microbiota of Patients with Gastric Cancer in Taiwan

**DOI:** 10.1038/s41598-017-18596-0

**Published:** 2018-01-09

**Authors:** Yung-Yu Hsieh, Shui-Yi Tung, Hung-Yu Pan, Chih-Wei Yen, Huang-Wei Xu, Ying-Jhen Lin, Yi-Fang Deng, Wan-Ting Hsu, Cheng-Shyong Wu, Chin Li

**Affiliations:** 1 Department of Gastroenterology and Hepatology, Chiayi Chang Gung Memorial Hospital, Chiayi, Taiwan; 2 College of Medicine, Chang Gung University, Taoyuan, Taiwan; 3 Department of Applied Mathematics, National Chiayi University, Chiayi, Taiwan; 4 Department of Life Science, National Chung Cheng University, Chiayi, Taiwan

## Abstract

*Helicobacter pylori* is recognised as a main risk factor for gastric cancer. However, approximately half of the patients with gastritis are negative for *H. pylori* infection, and the abundance of *H. pylori* decreases in patients with cancer. In the current study, we profiled gastric epithelium-associated bacterial species in patients with gastritis, intestinal metaplasia, and gastric cancer to identify additional potential pathogenic bacteria. The overall composition of the microbiota was similar between the patients with gastritis and those with intestinal metaplasia. *H. pylori* was present in half of the non-cancer group, and the dominant bacterial species in the *H. pylori*-negative patients were *Burkholderia*, *Enterobacter*, and *Leclercia*. The abundance of those bacteria was similar between the cancer and non-cancer groups, whereas the frequency and abundance of *H. pylori* were significantly lower in the cancer group. Instead, *Clostridium*, *Fusobacterium*, and *Lactobacillus* species were frequently abundant in patients with gastric cancer, demonstrating a gastric cancer-specific bacterial signature. A receiver operating characteristic curve analysis showed that *Clostridium colicanis* and *Fusobacterium nucleatum* exhibited a diagnostic ability for gastric cancer. Our findings indicate that the gastric microenvironment is frequently colonised by *Clostridium* and *Fusobacterium* in patients with gastric cancer.

## Introduction

The microbiota is an essential component of the human epidermal and mucosal environments. It is well recognised that specific microbes are associated with specific pathological conditions, especially in the alimentary tract where microbes are particularly abundant. These disease-associated microbes become more abundant in the microbiota under pathogenic conditions and are likely contribute to disease progression^1–3^. Such disease-promoting bacterial infections are best exemplified by the role of *Helicobacter pylori* in gastritis and gastric cancer^4,5^. Recent studies have also demonstrated that *Fusobacterium nucleatum* is enriched in colorectal cancer lesions and plays a role in promoting cancer invasiveness^6–8^. Hence, the colonisation of the alimentary tract by specific pathogenic microbes drives the development of gastrointestinal cancers.

The extreme acidity and thick protective mucosa of the gastric environment limit the growth and colonisation of bacteria. Therefore, the complexity of the gastric microbiota is generally much lower than that of the intestinal and oral microbiotas, and most of the gastric bacteria remain in the gastric juice^9^. Previous studies that have profiled the gastric microbiota have shown that *Streptococcus*, *Prevotella*, *Rothia*, *Porphyromonas*, and *Veillonella* are the most common bacterial genera. *Neisseria*, *Fusobacterium*, *Klebsiella*, and other potential pathogens have also been detected^10^. In addition, the composition of the microbiota is subject to rapid changes caused by food consumption. Indeed, most of the bacteria found in the gastric microbiota are undergoing passage from the oral cavity to the intestines^9^. However, *H. pylori* can penetrate the mucus layer to colonise the gastric mucosa and establish a long-term infection^5,11^. Once *H. pylori* establishes a growing colony under the mucosal layer, it produces urease and elevates the pH of its microenvironment^12^. *H. pylori* not only elicits a strong inflammatory response but also actively alters the cellular functions of the gastric mucosa^11,13^. This is accomplished by a type IV secretory machinery that is encoded by the *cag* pathogenicity island^14^. This system is used to deliver the virulence factors CagA and peptidoglycan to the gastric mucosal cells^15,16^. Once delivered to the mucosal cells, CagA is phosphorylated and modulates the structure and function of the cytoskeleton and cell-cell junctions, resulting in the disruption of mucosal integrity^15^. At the same time, peptidoglycan activates the PI3K-Akt signalling pathway to decrease apoptosis and promote cell migration^13,17^. Together, these virulence factors produced by *H. pylori* facilitate the transformation of gastric mucosal cells and lead to a drastic increase in the risk of gastric cancer.

Although *H. pylori* infection has been identified as the strongest risk factor for gastric cancer, only approximately half of all patients with gastritis are infected with *H. pylori* ^18,19^. Furthermore, only 1–2% of those *H. pylori*-positive patients eventually develop gastric cancer. Moreover, *H. pylori* often becomes undetectable in gastric cancer specimens^20–22^. This latter observation suggests that *H. pylori* infection is an early event to prime the gastric mucosa for further oncogenic changes. A hypothesis to account for the decline in the abundance of *H. pylori* in gastric cancer is microbial succession^10^. This hypothesis proposes that *H. pylori* creates a niche microenvironment on the gastric mucosa that facilitates its colonisation by secondary settler bacteria. It is reasonable to expect that *H. pylori* can be replaced as the predominant species in the growth niche. It is an often-considered hypothesis that other species of gastric microbes other than *H. pylori* also participate in promoting the development of gastric cancer^18,22^.

Here, we report an analysis of the gastric epithelium-associated microbiota in patients with gastritis and gastric cancer. Our results showed an underrepresentation of *H. pylori* among the gastric microbiota of the patients with gastric cancer, while *Fusobacterium* and *Clostridium* were frequently enriched. Therefore, our study provides evidence to support the phenomenon of microbial succession in the epithelium-associated microenvironment during gastric oncogenesis. The enrichment of *Fusobacterium* and *Clostridium* raises the possibility that those bacteria may be involved in gastric oncogenesis.

## Results

The gastric microbiota includes various passenger microbes undergoing transit from the oral cavity to the lower gut and resident bacteria closely associated with the gastric epithelium. *H. pylori*, which is one of the resident bacterial species, can induce chronic inflammation and promote host cell transformation. In this study, we aimed to analyse and compare the gastric microbiota that are closely associated with the gastric mucosa under distinct pathological conditions. To this end, we recruited patients who visited outpatient clinics and received an upper gastrointestinal endoscopic examination. Among the patients enrolled in our study, only two patients were classified as “normal” by pathologists, which is insufficient to represent a normal control in the statistical analysis. As the result, we did not include these two healthy subjects in subsequent analyses. Gastric biopsies collected during these examinations were reviewed by pathologists to determine the histological diagnosis of the lesions. At the same time, additional biopsies were collected for the analysis of the gastric epithelium-associated microbiota. According to their pathological reports, the patients enrolled were assigned to the gastritis, intestinal metaplasia, or cancer groups. The patients’ basic information is summarised in Table 1. For microbiota analysis, the biopsies were immediately subjected to extensive washing in phosphate-buffered saline several times to remove excess mucus and gastric fluid microbiota. Through this step, the microbes closely associated with the gastric tissues were expected to be enriched in the following metagenomic analysis. Host cell chromosomes and microbial genomic DNA were extracted together from the rinsed tissue specimens. The microbial ribosomal variable regions 3 and 4 were subsequently amplified and indexed using commercial reagents according to the manufacturer’s protocol (see Methods for details).

**Table 1:** Clinicopathological characteristics of the patient cohort.

Pathological condition	Age	Sex	CLO test	Giemsa staining	Lesion location	Lauren’s classification
Non-cancer						
gastritis	58	male	not done	positive	body	
	26	male	positive	positive	antrum	
	26	female	negative	positive	antrum	
	24	female	positive	negative	antrum	
	24	female	negative	negative	antrum	
	22	female	negative	negative	antrum	
	22	female	positive	positive	antrum	
	56	female	not done	negative	antrum	
	32	male	negative	negative	antrum	
Intestinal metaplasia	70	male	negative	negative	body	
	77	male	not done	positive	angle	
	51	male	positive	positive	antrum	
	34	male	negative	negative	antrum	
	44	female	positive	negative	antrum	
	24	female	negative	negative	antrum	
	24	female	negative	negative	antrum	
Cancer						
Stage I	71	male	not done	negative	body	intestinal
	85	female	not done	negative	antrum	n. d.
Stage II	53	male	not done	negative	antrum	diffuse
	77	male	not done	negative	antrum	n. d.
	75	female	positive	positive	antrum	intestinal
Stage III	75	male	not done	negative	antrum	n. d.
	62	male	not done	negative	antrum	diffuse
Stage IV	62	female	not done	negative	cardia	n. d.
	62	female	not done	negative	body	n. d.
	72	female	not done	negative	antrum	n. d.
	61	female	not done	negative	fundus	n. d.

Abbreviation: CLO test, Campylobacter-like organism test.

The sequencing of the indexed microbial ribosomal DNA replicons was performed using a MiSeq sequencer. The sequencing reads were subjected to rigorous quality control and then identified by comparing them with the NCBI microbial 16S ribosomal DNA database using the BLAST algorithm in CLC Genomic Workbench v.8.5. Most of the reads that were not matched to the databased microbial 16S ribosomal DNA sequences were human repetitive sequences and were removed from the subsequent analysis. In this study, we set the threshold for positive identification at 97% homology, and reads with lower than 97% homology to the closest reference bacterial species were reported as unclassified bacteria. The reads that were reported as different strains or subtypes of the same species were combined to calculate the abundance of each bacterial species. The sequencing reads were also analysed using the USEARCH package, and we found no statistical difference between the results obtained by BLAST and those obtained by the USEARCH algorithms. Since the microbial species identified by these two algorithms were nearly identical, we used the result of BLAST analysis for subsequent statistical analysis. The abundance of each microbial species in the microbiota was represented as the percentage of total reads. We arbitrarily used 1% of the total reads as a threshold to define the abundance of each bacterial species. Bacteria occupying less than 1% reads in all specimens were defined as low-abundance species, while those occupying more than 1% reads in at least one specimen were defined as high-abundance species. When the combination of bacterial species in a genus was greater than 1% of the reads, the genus was defined as a high-abundance genus (Supplementary Table 1). An inspection of our data shows drastic differences in the number and species of high-abundance microbes between specimens.

**Figure 1:**
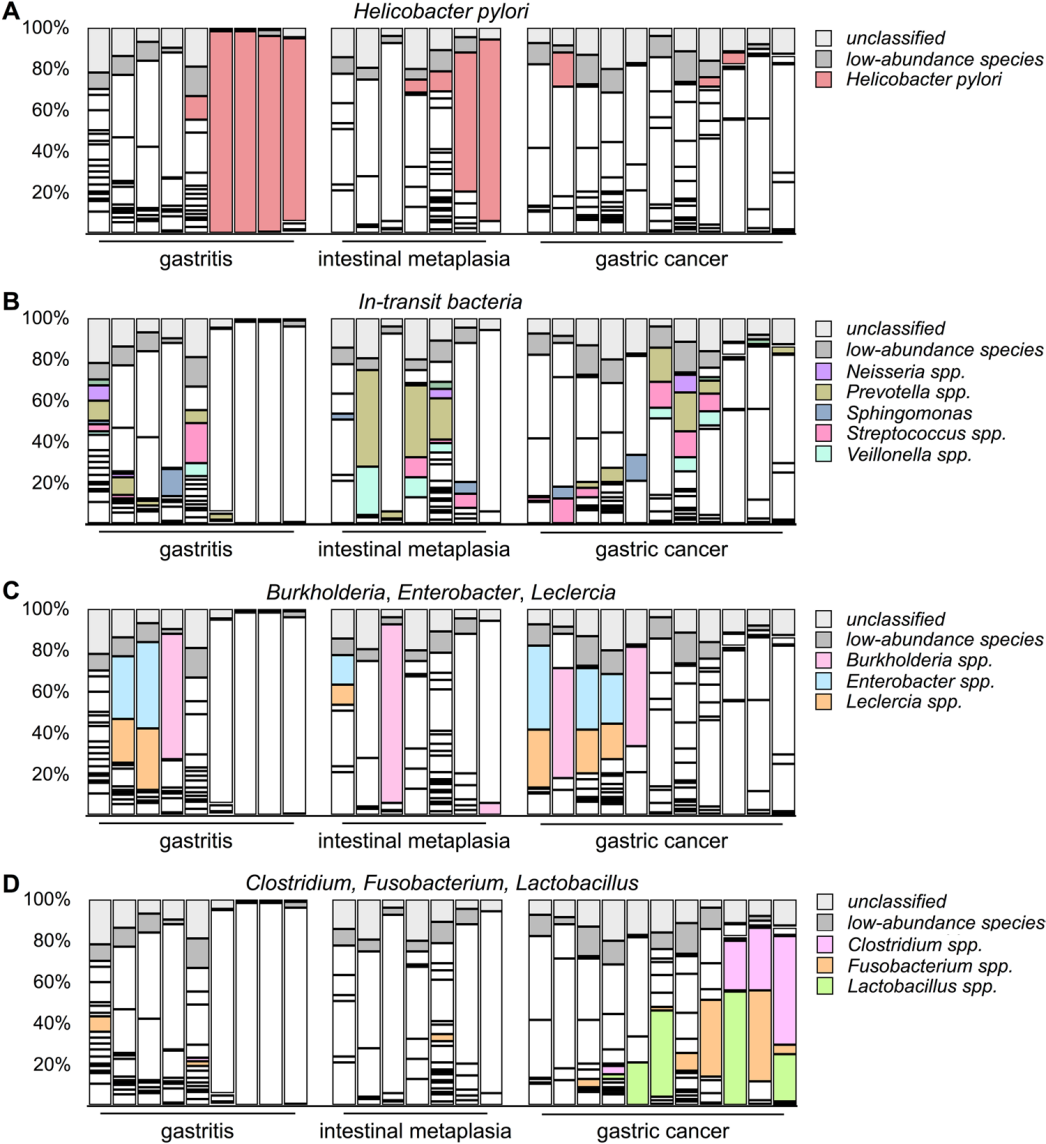
Identification of changes in the composition of the microbiota between patients with gastric cancer and those with other gastrointestinal disorders by 16S ribosomal DNA metagenomic analysis. (**A**) The abundance of *Helicobacter pylori* in the patients’ microbiota. (**B**) The proportions of high-abundance in-transit microbes. (**C**) The abundance of *Burkholderia*, *Enterobacter*, and *Leclercia*. (**D**) The abundance of *Clostridium*, *Fusobacterium*, and *Lactobacillus*.

We first investigated the prevalence of *H. pylori* infection in our study cohort. In the gastritis group, *H. pylori* was identified as a high-abundance species in 5 of 9 patients. Strikingly, in 4 of the 5 patients with *H. pylori*-positive gastritis, *H. pylori* occupied more than 90% of the microbiota (Fig. 1A), indicating a *H. pylori-*predominant microbiota on the gastric epithelium. In the intestinal metaplasia group, *H. pylori* was observed in 4 of 7 metaplasia patients, and *H. pylori* dominance was observed in 2 patients. A statistical analysis showed no difference in the frequency of *H. pylori* infection between the patients with gastritis and those with intestinal metaplasia. The results indicated that the epithelium-associated microbiota in approximately 60% of the patients with non-cancer conditions was dominated by *H. pylori*. This observation also indicated that the *H. pylori* infection rate among the non-cancer groups was similar to that in previous reports. In contrast to the non-cancer group, the prevalence of *H. pylori* was clearly much lower in the cancer group. Specifically, *H. pylori* was detected as a high-abundance species in only 3 of 11 specimens, and none of those patients exhibited an extreme dominance of *H. pylori* as was observed in several of the patients with non-cancer gastric disorders (Fig. 1A). Taken together, our findings showed that the prevalence of *H. pylori* was significantly lower in the gastric mucosa-associated microbiota of patients with gastric cancer than in that of patients with non-cancer gastric disorders.

In comparison with the microbiota in the *H. pylori-*positive patients, that in the *H. pylori-*negative patients appeared to be more diverse. The bacterial species in the *H. pylori-*negative patients with gastritis belonged to the genera *Campylobacter*, *Neisseria*, *Prevotella*, *Sphingomonas*, *Streptococcus*, and *Veillonella* (Fig. 1B). The same bacterial species were also found in the intestinal metaplasia and cancer groups with a similar frequency, except for an increased level of *Prevotella* in some patients. A statistical analysis showed no significant difference in the prevalence of these in-transit microbes between the non-cancer and cancer groups. These bacteria are known components of the gastric fluid microbiota^9^ and were thought to be undergoing transit to the lower part of the alimentary tract. We speculated that the number of gastric mucosa-attached bacteria was relatively low in the *H. pylori-*negative patients. As a result, despite our efforts to remove loosely-bound microbes during sample preparation, the remaining in-transit microbes still occupied a significant portion of the sequencing libraries. Consequently, these bacteria were identified as high-abundance species in *H. pylori-*negative specimens. Since these bacteria were not specifically enriched in the cancer-associated microbiota, we assumed that these microbes do not play a role in gastric inflammation or cancer development.

Besides the previously known in-transit bacteria, several other species were identified as high-abundance species in the *H. pylori*-negative patients. These bacteria were not part of the typical gastric juice microbiota, and their abundance suggests that they are likely bona-fide colonisers on the gastric mucosa. Some of these microbes, including *Burkholderia, Enterobacter*, and *Leclercia*, were identified in both the non-cancer and cancer groups (Fig. 1C), and a statistical analysis showed a similar prevalence in all patient groups. It was reported that an increased level of *Enterobacter* was present in the gastric fluid of patients with esophagitis and Barrett’s oesophagus^23^, suggesting that *Enterobacter* may cause mucosal inflammation. The most abundant *Burkholderia* and *Leclercia* species identified in the present study were *Burkholderia fungorum* ^24,25^ and *Leclercia adecarboxylata* ^26,27^. Both of these species are opportunistic pathogens of epithelial and mucous tissues, but their capability to infect the gastric mucosa has not been reported before. Since the presence of these microbes and *H*. *pylori* in the microbiota appeared to be mutually exclusive in the patients with gastritis, it seems possible that *Burkholderia*, *Enterobacter*, and *Leclercia* can colonise the gastric mucosa as *H. pylori* does.

In addition to the bacteria found in all patient groups, we also discovered specific high-abundance bacterial species in more than half of the patients with cancer. These bacteria belonged to the genera *Clostridium*, *Fusobacterium*, and *Lactobacillus* (Fig. 1D). The abundance and prevalence of *Clostridium* and *Fusobacterium* in the cancer specimens were higher than those of *Lactobacillus*. Among these bacteria, *F. nucleatum* has been clearly shown to play a role in development of colorectal cancer^6,7,28^. Hence, our result suggests a possibility that *Fusobacterium* may also participate in gastric oncogenesis. Another bacterial genus that was enriched in the gastric cancer specimens was *Clostridium*. Although several species of *Clostridium* are well-known pathogens, the most abundant *Clostridium* species identified in this study was *Clostridium colicanis* ^29,30^. Although this species has already been identified, its pathogenicity toward humans has not been studied before. On the other hand, we also found that *Lactobacillus* was a high-abundance species in patients with cancer. Traditionally considered as in-transit probiotic bacteria, the colonisation of the gastric mucosa by *Lactobacillus* suggests a drastic change in the gastric microenvironment of patients with cancer. In summary, our data showed that various bacteria were specifically enriched in gastric cancer tissues. It is possible that the colonisation of the gastric mucosa by these bacteria is only a consequence of cancer development. However, given that at least some of these bacteria are pathogenic, it is also possible that these bacteria contribute to the development or progression of gastric cancer.

**Figure 2:**
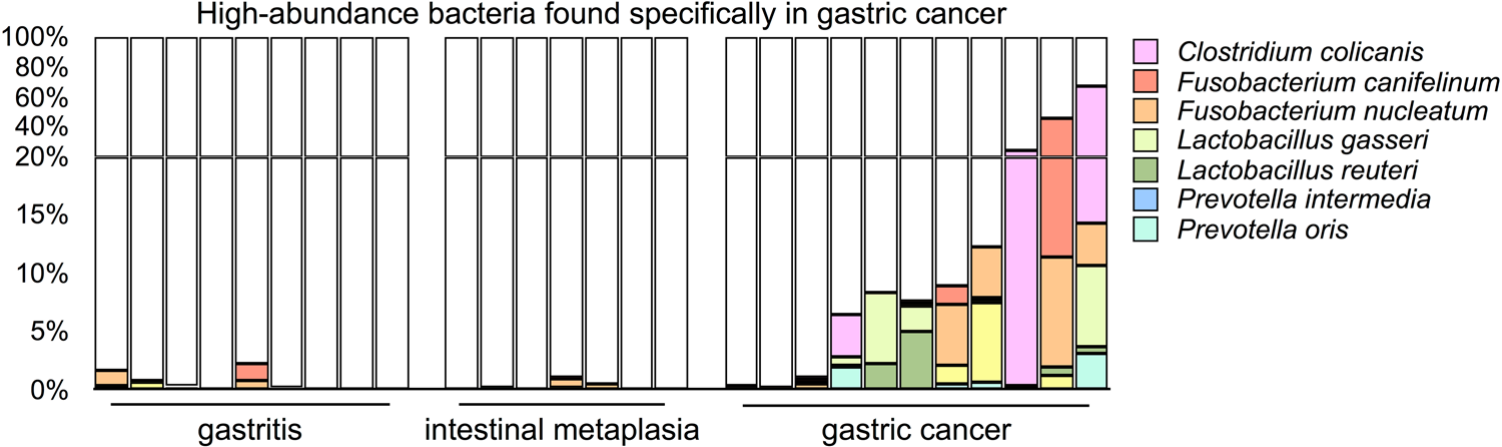
Bacteria specifically enriched in the gastric cancer-associated microbiota.

We also performed a further statistical analysis of the BLAST data using the DESeq. 2 package^31^. The analysis at the species level revealed that 47 microbial species exhibited significant differences in abundance between the gastritis and cancer patient groups. Besides a decrease in the abundance of *H. pylori* in the cancer patient, 10 high-abundance strains were specifically enriched in the majority of the cancer group (Table 2). Five of these species, including *C. colicanis*, *Fusobacterium canifelinum* ^31^, *F. nucleatum*, *Lactobacillus gasseri* ^32^, and *Lactobacillus reuteri* ^33,34^, were highly abundant in multiple cancer specimens. In addition, we also identified an increased abundance of *Prevotella intermedia* ^35,36^ and *Prevotella oris* ^37^ in the cancer-associated microbiotas despite the combined percentage of *Prevotella* spp. appearing to show no significant difference among the patient groups (Fig. 1B). Among these seven species, *C. colicanis*, *L. gasseri*, and *L. reuteri* were found only in the cancer group while *F. canifelinum* and *F. nucleatum* were also detected in the non-cancer group with a low frequency. Collectively, the abundance of *C. colicanis*, *F*. *canifelinum*, *F*. *nucleatum*, *L. gasseri*, *L*. *reuteri*, *P. intermedia*, and *P. oris* constituted more than 5% of microbiota in 8 of 11 patients with gastric cancer (Fig. 2). Hence, our finding demonstrates that most of the patients with gastric cancer exhibited a distinct gastric microbiota from that of the patients with non-cancer gastric disorders.

**Table 2:** Bacteria specifically found in the patients with gastric cancer.

Strain	Average counts of cancer specimens	Log2 fold change*	*p*-value	Adjusted *p*-value
*Clostridium colicanis*	2063.74	8.16	0.0000	0.0000
*Fusobacterium canifelinum*	173.77	4.16	0.0001	0.0009
*Fusobacterium nucleatum*	2251.23	3.24	0.0022	0.0142
*Lactobacillus gasseri*	270.50	6.34	0.0000	0.0000
*Lactobacillus reuteri*	332.55	6.51	0.0000	0.0000
*Megasphaera micronuciformis*	212.038	3.49	0.0010	0.0076
*Prevotella intermedia*	881.99	3.14	0.0063	0.0313
*Prevotella oris*	698.158	5.52	0.0000	0.0000
*Streptococcus gordonii*	123.70	5.58	0.0000	0.0000
*Streptococcus parasanguinis*	142.09	4.56	0.0000	0.0000

*Compared with the average counts of the gastritis specimens.

**Figure 3:**
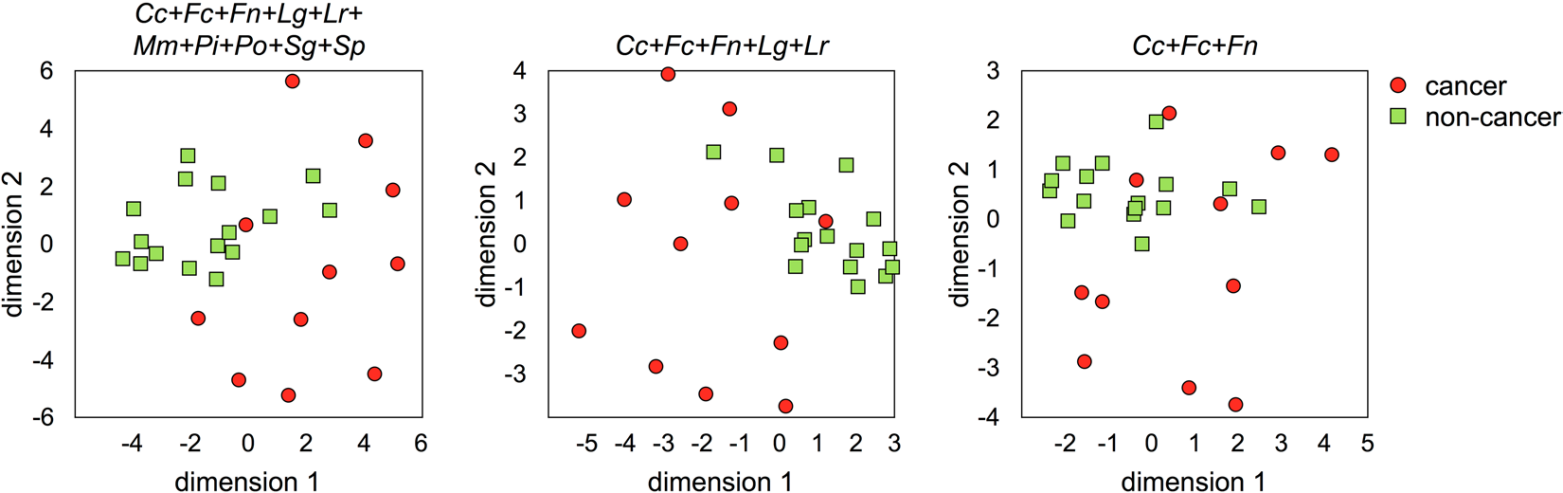
Identification of the bacterial signature of gastric cancer. Multidimensional analyses were performed considering various combinations of the following bacterial species: *Clostridium colicanis (Cc), Fusobacterium canifelinum (Fc), Fusobacterium nucleatum (Fn), Lactobacillus gasseri (Lg), Lactobacillus reuteri* (*Lr*), *Megasphaera micronuciformis* (*Mm*)*, Prevotella intermedia* (*Pi*), *Prevotella oris* (*Po*), *Streptococcus gordonii* (*Sg*), and *Streptococcus parasanguinis* (*Sp*).

To investigate whether there is a representative bacterial signature for gastric cancer, we performed multidimensional analyses considering various combinations of the detected bacterial species. When all the species of the microbiota were included in the analysis, no discernible pattern could be observed among the patient groups. The results remained inconclusive when 47 cancer-enriched bacterial species were used in the analysis. However, when ten high-abundance bacteria (listed in Table 2) were included in the analysis using the Euclidean distance metrics, we were able to distinguish all but one of the cancer specimens from the non-cancer specimens (Fig. 3). We then attempted to reduce the number of the bacterial species used to define this cancer-associated signature. Our tests showed that considering the combined abundance of *C. colicanis*, *F. canifelinum*, *F. nucleatum*, *L. gasseri*, and *L. reuteri* was sufficient to distinguish between non-cancer and cancer specimens (Fig. 3). Further reducing the number of bacterial species in the analysis generated a signature comprising only *C. colicanis*, *F. canifelinum*, and *F. nucleatum*. This three-species signature appeared to be present in more than half of the cancer specimens (Fig. 3). Based on this analysis, we think that it may be possible to use these cancer-enriched bacteria for gastric cancer diagnosis.

**Figure 4:**
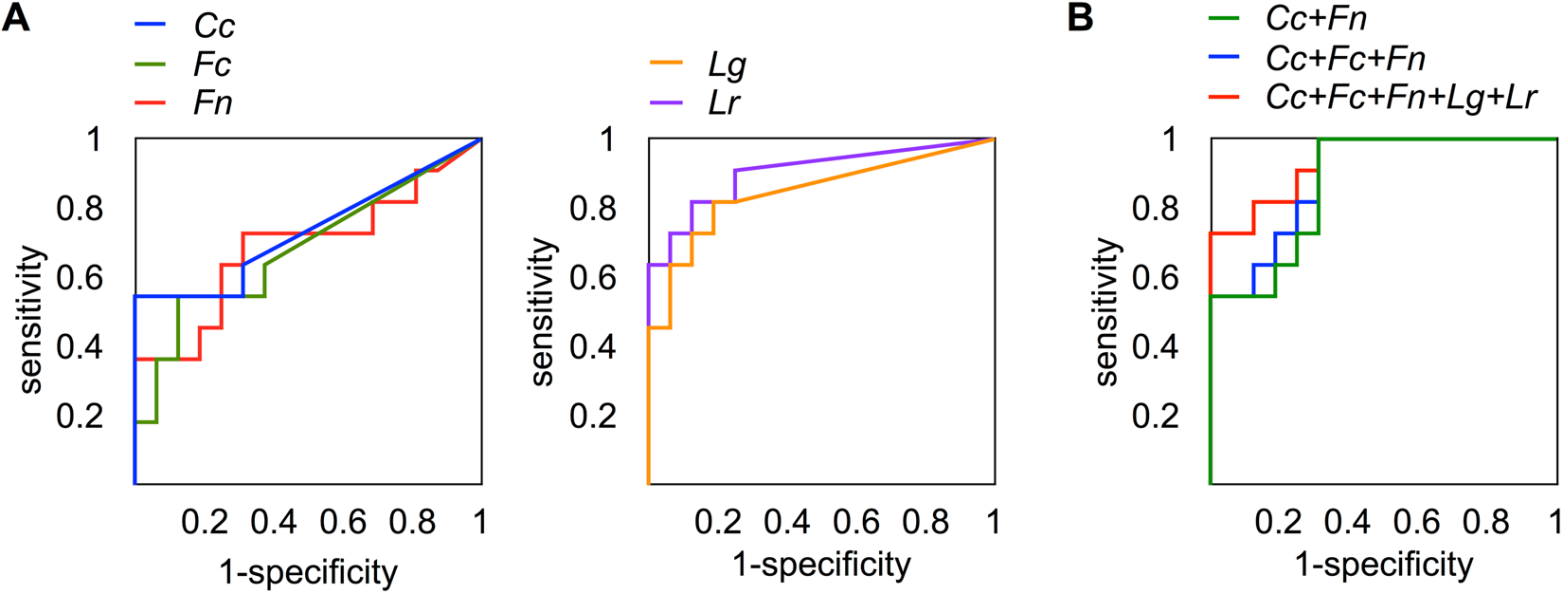
Evaluation of the discriminatory power of using bacterial abundance as a diagnostic tool. (**A**) The discriminatory power of the abundance of each individual species *C. colicanis*, *F. canifelinum*, *F. nucleatum*, *L. gasseri*, and *L. reuteri* was tested by a receiver operating characteristic curve analysis. (**B**) The combined abundance of *C. colicanis* and *F. canifelinum* (*Cc* + *Fn*), *C. colicanis*, *F. canifelinum*, and *F. nucleatum* (*Cc* + *Fc* + *Fn*), and *C. colicanis*, *F. canifelinum*, *F. nucleatum*, *Lactobacillus gasseri*, and *Lactobacillus reuteri* (*Cc* + *Fc* + *Fn* + *Lg* + *Lr*) was examined.

To determine whether the identified bacterial signature can be used as a diagnostic tool for gastric cancer, a receiver operating characteristic curve analysis was performed. We first examined the prediction power of single bacterial species using the abundance of *C. colicanis, F. canifelinum, F. nucleatum*, *L. gasseri*, or *L. reuteri*. The abundance of these bacterial strains in each patient is listed in Supplementary Table 2. The results showed that *C. colicanis, F. canifelinum*, and *F. nucleatum* exhibited an insufficient distinguishing power to identify gastric cancer. In contrast, *L. gasseri*, and *L*. *reuteri* showed an excellent sensitivity with a high area under the curve (Fig. 4A). We then investigated whether a combination of these bacteria could demonstrate a better prediction ability. An analysis using a combination of five species resulted in 73% sensitivity and 100% specificity. In contrast, the combination of *C. colicanis, F. canifelinum*, and *F. nucleatum* exhibited 100% sensitivity but approximately 70% specificity (Fig. 4B). Interestingly, a nearly identical result could be achieved using only *C*. *colicanis* and *F. nucleatum* in the receiver operating characteristic analysis. This result reflects the observation that *F. canifelinum* and *F. nucleatum* were almost always detected together in the specimens. The performance of the bacteria as a diagnostic tool for gastric cancer is summarised in Table 3. Together, our data indicated that *C. colicanis* and *F. nucleatum* were highly abundant in the cancer specimens and could positively identify gastric cancer with 100% sensitivity. Hence, our findings suggest that *C. colicanis* and *F. nucleatum* might represent diagnostic markers for the detection of gastric cancer.

**Table 3:** Performance of individual bacterial species and combinations of bacterial species for gastric cancer diagnosis.

	Cc	Fc	Fn	Lg	Lr	Cc + Fn	Cc + Fc + Fn	Cc + Fc + Fn + Lg + Lr
AUROC	73.3%	68.2%	68.8%	84.7%	90.3%	87.5%	89.2%	93.8%
SE	0.108	0.111	0.112	0.084	0.065	0.066	0.060	0.044
p-value	0.043	0.114	0.103	0.003	<0.001	0.001	0.001	<0.001
95% CI	0.52–0.95	0.47–0.90	0.47–0.91	0.68–1.011	0.78–1.03	0.75–1.00	0.77–1.01	0.85–1.02
Cut-off	9.21 × 10^−4^	2.00 × 10^−4^	1.10 × 10^−3^	5.07 × 10^−6^	2.53 × 10^−5^	1.12 × 10^−3^	1.22 × 10^−3^	3.86 × 10^−2^
Sensitivity	45.5%	54.5%	72.7%	81.8%	81.8%	100%	100%	72.7%
Specificity	0.00%	87.5%	68.8%	81.3%	87.5%	68.8%	68.8%	100%
PPV	23.8%	75.0%	61.5%	75.0%	81.8%	68.8%	68.8%	100%
NPV	0.00%	73.7%	78.6%	86.7%	87.5%	100%	100%	84.2%

Abbreviations: AUROC, area under the receiver operating characteristics curve; CI, confidence interval; NPV, negative predictive value; PPV, positive predictive value; SE, standard error. Bacterial species: *Cc, Clostridium colicanis; Fc, Fusobacterium canifelinum; Fn, Fusobacterium nucleatum; Lg, Lactobacillus gasseri; Lr, Lactobacillus reuteri*.

## Discussion

The risk of gastric cancer is greatly increased by viral and bacterial infection. Although the risk of *H. pylori* infection in gastric cancer is clearly understood, the role of other gastric bacteria remains to be investigated. Various experimental approaches have been employed to explore the microbiota in patients with gastric cancer. An early study using the terminal restriction fragment length polymorphism technique showed a decreased abundance of *H. pylori* and an enrichment of *Streptococcus*, *Lactobacillus*, *Veillonella*, and *Prevotella* ^38^. Gastric microbiota profiling of gastric cancer using microarrays reported a decrease in bacterial diversity in gastric cancer compared with that in gastritis^19^. With the advance in sequencing technology, various sequencing strategies and platforms were employed to profile gastric cancer-associated microbiota. Two independent studies using pyrosequencing to analyse Korean patient cohorts showed the abundance of *H. pylori* decreases in gastric cancer. As a result, the microbiota diversity increases in gastric cancer patients if compared with *H. pylori*-dominant gastritis patients^20,39^. An additional study analysing a large Hong Kong patient cohort also showed that *H. pylori* infection decreases in gastric cancer patients^40^. Eradication of *H. pylori* will lead to an increase in microbiota complexity^40^. An independent study by Yu and co-workers on cohorts from China and Mexico showed that the abundance of *H. pylori* is lower in gastric lesions than paired non-malignant controls^41^. Hence, current evidence support the notion that the abundance of *H. pylori* decreases in gastric cancer patients. On the other hand, due to the great variation between study cohorts, there is no consistent finding on other predominant bacteria in the cancer-associated microbiota.

In this study, we analysed the microbiota composition in patients with gastric cancer, gastritis, and intestinal metaplasia by sequencing variable regions 3 and 4 of the 16S ribosomal RNA gene. Despite using a different profiling technique compared with those employed in previous studies, our data indicated that the abundance of *H. pylori* was lower in the patients with gastric cancer than in the other patient groups, which is largely consistent with previous reports. We also found an increased abundance of *Clostridium*, *Fusobacterium*, and *Lactobacillus* in gastric cancer tissues. *Lactobacillus* is a facultative anaerobe and represents a component of the gut microbiota, while *Fusobacterium* and *Clostridium* are strict anaerobes and represent components of the oral cavity microbiota. Since these bacteria apparently enter the gastric microenvironment through dietary intake, it is reasonable to assume that these bacteria opportunistically infect the gastric epithelium during their transit to the alimentary tract. More importantly, it is necessary to determine whether the variation of the gastric microbiota composition is a driving event of gastric cancer development. A recent study by Coker and co-workers analysing cohorts from China and Mongolia identified a gastric cancer-specific microbial signature^42^. This signature includes *Peptostreptococcus stomatis, Streptococcus anginosus, Parvimonas micra, Slackia exigua*, and *Dialister pneumosintes* ^42^. Distinct bacteria signatures in gastric microbiota may be a reflection of multiple environmental factors, dietary customs, and health care accessibility. Given the complexity of contributing factors, it may be necessary to determine how each of these factors affects gastric microbiota in order to consolidate the findings made in independent studies.

One of the enriched strains in the gastric cancer microbiota is *F. nucleatum*, which has also been reported to be enriched in the colorectal cancer microbiota^8,43,44^. *F. nucleatum* attaches to cancer tissues through the interaction of *Fusobacterium* lectin Fap2 with tumour-specific surface Gal-Gal NAc, and this interaction leads to MUC2 and TNFα expression in the colon cancer cells^44,45^. Clinically, the level of *F. nucleatum* DNA is correlated with a poor patient prognosis^10^. Together, the current evidence strongly suggests a role of *F. nucleatum* in promoting the growth and metastasis of colorectal cancer. The detection of *Fusobacterium* DNA by polymerase chain reaction (PCR) has been used to increase the detection sensitivity of standard faecal immunochemical tests^46^, indicating its potential clinical application as a non-invasive diagnostic marker. Given its importance in colorectal cancer, it is possible that *F. nucleatum* also plays a role in gastric cancer. In addition, it may be necessary to eradicate *Fusobacterium* infection to prevent or retard alimentary tract-related cancer progression.

Another bacterial species that was abundant in the cancer microbiota in the present study is *C. colicanis*. The draft genome sequence of *C. colicanis* indicated that it is a distinct subgroup of *Clostridium* and can reduce nitrate to nitrite^47^. Its role in human disease is completely unknown, and further study is warranted. The most unexpected aspect of our findings was an increased abundance of *Lactobacillus* in the cancer-associated microbiota. *Lactobacillus* is a facultative anaerobe and represents a component of the gut microbiota, and it is generally considered to be a probiotic in-transit passenger. A possible explanation for our observations may be that the increased abundance of *Lactobacillus* resulted from the dietary and medical use of probiotic microbes among the patients with gastric cancer. This is because the use of probiotic microbes as a dietary supplement is believed to be able to relieve various gastrointestinal conditions^48^. It is a rather common clinical practice in Taiwan to prescribe probiotic tablets for patients with gastrointestinal disorders, and probiotic tablets are also available over-the-counter in drug stores. Since the number of probiotic microbes per dosage is extremely high, a residue of the dosage could represent a significant part of the microbiota. However, a review of the patients’ medical history showed that no probiotic tablets were prescribed for any patient in our cohort, although we cannot exclude the possibility of the use of over-the-counter probiotic tablets. Alternatively, the increased association of *Lactobacillus* with the gastric mucosa in patients with gastric cancer may indicate that it can become a sedentary resident of the gastric microbiota as a result of microenvironment change. At present, we cannot distinguish between these two possibilities. Additional investigation is needed to determine the underlying mechanism.

Approximately half of the non-cancer group had a severe *H. pylori* infection, but the prevalence and abundance of *H. pylori* drastically decreased in the cancer-associated microbiota. We speculated that the succession of microbial species is the likely cause of those observations. Since the prevalence of other microbial species showed no significant difference between the non-cancer and cancer groups, there is a distinct possibility that *H. pylori* is replaced by *Clostridium, Fusobacterium*, and *Lactobacillus*. Assuming that *H. pylori* acts as the first invader of the gastric mucosa, it would change the microenvironment in such a way to create a growth niche that allows for its persistent infection. This growth niche may be also open for the colonisation of secondary settler bacteria. It is likely that only a few bacteria from the oral cavity can outcompete and replace *H. pylori*. Alternatively, *H. pylori* may be eliminated by the host immune response, leaving the growth niche to be occupied by opportunistic pathogens. Regardless of how *H. pylori* is removed from the growth niche, our data indicate that *Clostridium*, *Fusobacterium*, and *Lactobacillus* are the most common secondary successors.

The microbial succession event may occur before or after cancer development. However, if the secondary settler bacteria possess an oncogenic potential, the colonisation of the gastric mucosa by these microbes may facilitate the oncogenic transformation of the gastric epithelium. In this scenario, the secondary settler bacteria may collaborate with *H. pylori* to drive gastric cancer development. One such candidate bacterial species identified in our study is *F. nucleatum*. Alternatively, the succession event may be the result of a microenvironment change resulting from the development of cancer. These two possibilities are not mutually exclusive; some microbes may act as cancer drivers while the colonisation of the gastric mucosa by other microbes may represent a passenger event of cancer development. Hence, it is essential to delineate the relationship of *H. pylori* with the cancer-enriched bacterial species identified in our study. Further studies will provide a fundamental understanding of whether collaboration among infectious bacteria promotes gastric cancer formation with a higher efficiency.

Regardless of the roles of *Clostridium*, *Fusobacterium*, and *Lactobacillus* in oncogenesis, the enrichment of these microbes serves as a bacterial signature of gastric cancer. Our data showed that the abundance of *C. colicanis* and *F. nucleatum* exhibits an excellent predictive ability and represents a potential diagnostic marker. However, gastric microbial analysis is not currently a feasible alternative approach to traditional diagnostic or screening methods. Unlike faecal specimens, which can be used as a non-invasive screening method for colorectal cancer, there is currently no easy method to collect the gastric microbiota. Our analysis is still dependent on the acquirement of biopsies through upper gastrointestinal tract endoscopic examination. However, if gastric biopsies are already available for histological examination, there is no need for any other type of diagnostic procedure. Thus, it is necessary to have a non-invasive method to collect the gastric microbiota before bacterial analysis can be applied as a diagnostic tool. Nevertheless, our study on the cancer-associated gastric microbiota provides a foundation for a more comprehensive understanding of the pathogenic bacteria present in the gastric microbiota. If any bacterial species is found to participate in promoting gastric cancer, the eradication of this bacteria should be beneficial to the patients and may provide a means to further decrease the prevalence of gastric cancer.

## Methods

### Patient cohort

Gastric biopsies were collected during upper gastroenterology endoscopic examination. The acquisition and use of clinical specimens in this study were performed in accordance with the Declaration of Helsinki, and all the patients participating in this study were clearly informed and signed written informed consent. This study was approved and overseen by the Institutional Review Board of Chiayi Chang Gung Memorial Hospital (institutional review board approval numbers 102-2002B and 102-2004B).

### DNA extraction

Biopsies were rinsed extensively in phosphate-buffered saline to remove the mucus. Rinsed specimens were immersed overnight in RNAlater™ reagent (Thermo Fisher Scientific, Waltham, MA, USA) and stored at −80 °C. Biopsies were ground in TRI Reagent^®^ (Thermo Fisher Scientific) and centrifuged to remove undissolved debris. Total DNA, including both cellular and microbial DNA, was extracted according to the manufacturer’s protocol. The concentration of DNA was determined by fluorometric quantification.

### Bacterial 16S ribosomal DNA analysis

The protocol for 16S ribosomal DNA analysis was modified from the manual supplied by the manufacturer (Illumina, San Diego, CA, USA). Briefly, variable regions 3 and 4 of the bacterial 16S ribosomal RNA gene were amplified from purified DNA specimens. The degenerate primers for annealing to the conserved bacterial 16S rRNA gene sequences were adapted from the previous report. To increase the sequencing efficiency and data quality, we used a set of mixed primers with one to three nucleotides placed between the annealing and adaptor sequences of the primers. The sequences of the primers are given in Table 4. The PCR products were separated by electrophoresis in an agarose matrix, and the products with the expected sizes were purified from the matrix. Addition of the index and sequencing sequences was performed by second-stage PCR using a Nextera^®^ XT index kit (Illumina). Sequencing-ready libraries were analysed by capillary electrophoresis and quantified by a fluorescence-based method. Sequencing was performed in a MiSeq sequencer (Illumina) for 18 dark cycles and 350 read cycles in the forward read and 18 dark cycles and 250 read cycles in the reverse read.

**Table 4:** The primer sequences for amplifying variable regions 3 and 4 of the bacterial 16S ribosomal DNA sequences.

	Adaptor sequence	Spacer	Annealing sequence
Forward primer	5′-TCGTCGGCAGCGTCAGATGTGTATAAGAGACAG		CCTACGGGNGGCWGCAG
	5′-TCGTCGGCAGCGTCAGATGTGTATAAGAGACAG	A	CCTACGGGNGGCWGCAG
	5′-TCGTCGGCAGCGTCAGATGTGTATAAGAGACAG	TD	CCTACGGGNGGCWGCAG
	5′-TCGTCGGCAGCGTCAGATGTGTATAAGAGACAG	GDR	CCTACGGGNGGCWGCAG
Reverse primer	5′-GTCTCGTGGGCTCGGAGATGTGTATAAGAGACAG		GACTACHVGGGTATCTAATCC
	5′-GTCTCGTGGGCTCGGAGATGTGTATAAGAGACAG	T	GACTACHVGGGTATCTAATCC
	5′-GTCTCGTGGGCTCGGAGATGTGTATAAGAGACAG	AC	GACTACHVGGGTATCTAATCC
	5′-GTCTCGTGGGCTCGGAGATGTGTATAAGAGACAG	GTT	GACTACHVGGGTATCTAATCC

The paired-end sequencing reads were trimmed using Q20 as a threshold, and the forward and reverse reads were merged. Non-merged reads and merged reads shorter than 390 nucleotides were discarded. Trimmed and filtered reads were identified by BLAST searches against the NCBI microbial 16S database using CLC Genomic Workbench v.8.5. The reads with no match were reported as unidentified, while the reads with lower than 97% homology to the best-matched sequences were reported as unclassified. The results were exported into R for further statistical analysis. The reads were also identified through the operational taxonomical unit approach using the USEARCH package.

### Statistical analysis

Significant differences in the abundance of the microbial strains among the patient groups were calculated using the DESeq. 2 package in R. A multidimensional analysis was performed for quality assessment and to explore the relationships among specimens, based on Euclidean distances calculated from the regularised-logarithm transformed counts. A receiver operating characteristic (ROC) curve analysis was performed to evaluate the diagnostic value of the bacterial candidates for distinguishing gastric cancer specimens from non-cancer specimens. Both analyses were performed using the SAS Enterprise 5.1 statistical package (SAS Institute, Cary, NC, USA). A conventional level of significance (*p* < 0.05) was used for rejecting the null hypothesis. To determine the bacteria with discriminating ability to differentiate cancer from non-cancer specimens, we first detected the significance difference of microbiota from gastric cancer and non-cancer patients using DESeq. 2 package. Next, a ROC curve was used to assess the diagnostic value of bacterial candidates in distinguishing gastric cancer and controls. The best cut-off values were determined by ROC analyses that maximized the Youden index (Sensitivity + Specificity − 1).

### Data availability

The data files have been deposited in the NCBI Sequence Read Archive. The Bioproject accession number is PRJNA387097. The Biosample accession numbers are SAMN07138993, SAMN07138994, SAMN07138995, SAMN07138996, SAMN07138997, SAMN07138998, SAMN07139000, SAMN07139001, SAMN07139002, SAMN07139003, SAMN07139004, SAMN07139005, SAMN07139006, SAMN07139007, SAMN07139008, SAMN07139009, SAMN07139010, SAMN07139013, SAMN07139013, SAMN07139015, SAMN07139016, SAMN07139017, SAMN07139018, SAMN07139019, SAMN07139020, SAMN07139024, and SAMN07139025.

## Electronic supplementary material

Supplementary Information
