# Supplementary material for: Increased Abundance of Clostridium and Fusobacterium in Gastric Microbiota of Patients with Gastric Cancer in Taiwan
Source: Sci Rep. 2018 Jan 9;8:158. doi: 10.1038/s41598-017-18596-0 (PMC5760541; doi:10.1038/s41598-017-18596-0)
Supplement: Supplementary file 1 — Supplementary Information [file 41598_2017_18596_MOESM1_ESM.pdf]

Supplementary data

Increased Abundance of *Clostridium* and *Fusobacterium* in Gastric Microbiota of Patients with Gastric Cancer in Taiwan

Yung-Yu Hsieh, Shui-Yi Tung, Hung-Yu Pan, Chih-Wei Yen, Huang-Wei Xu, Ying-Jhen Lin, Yi-Fang Deng, Wan-Ting Hsu, Cheng-Shyong Wu, Chin Li

Table S1. The percentages of bacteria identified by 16S ribosomal DNA sequencing

genus	gastritis									intestinal metaplasia							cancer										
unclassified	21.48%	13.66%	7.10%	9.96%	18.61%	4.31%	0.67%	0.64%	0.95%	14.41%	19.34%	3.88%	20.26%	11.14%	4.48%	5.48%	7.38%	8.65%	13.38%	20.16%	16.87%	4.23%	11.61%	16.23%	11.64%	8.28%	12.33%
low abundance	8.19%	9.08%	8.92%	2.18%	14.78%	1.03%	1.23%	0.83%	3.11%	7.96%	5.58%	3.81%	4.89%	9.97%	7.47%	0.36%	10.26%	3.36%	13.91%	11.12%	1.46%	9.87%	14.41%	7.73%	0.64%	1.78%	0.94%
Acinetobacter										21.07%				2.09%													
Aeribacillus	11.00%	5.53%	6.23%		3.32%				1.33%	3.02%				3.44%	2.72%		10.67%		6.76%	5.64%			2.04%				
Alloprevotella	4.95%	2.53%				0.31%																	1.25%				
Bacillus	1.21%																			1.05%							
Bacteroides																				1.37%							
Bifidobacterium																											0.12%
Bradyrhizobium					3.60%																		1.57%				
Brevundimonas	2.20%																										
Burkholderia				60.19%								85.88%				6.09%		52.95%			47.89%						
Campylobacter	3.04%	1.57%				0.34%								3.55%									1.41%	1.54%	0.11%	2.35%	
Caulobacter				1.45%																							
Clostridium					1.74%															3.67%					24.10%	30.46%	52.18%
Corynebacterium				7.09%										1.04%													
Cupriavidus														2.08%													
Enterobacter		30.29%	41.59%			0.28%				14.31%							40.42%		29.81%	23.89%					0.25%		0.14%
Enterococcus																				1.27%							
Escherichia	1.25%										3.31%				2.66%		1.00%		1.00%	2.13%							
Eubacterium																										1.41%	
Fusobacterium	7.66%	1.37%			2.13%	0.49%								3.44%					3.52%			37.28%	8.31%	1.63%	0.42%	43.59%	4.55%
Granulicatella					1.17%																						
Haemophilus	3.60%	2.00%				0.22%						2.03%	13.09%	3.01%	2.67%								1.82%	1.09%	0.15%		1.02%
Halomonas	3.03%	1.15%			1.72%									1.18%						1.49%							
Helicobacter					10.94%	88.61%	98.10%	98.53%	94.62%				6.18%	9.66%	67.54%	88.07%		16.72%	1.03%					4.73%	5.44%		
Lachnoanaerobaculu						0.14%																					0.11%
Lactobacillus																				2.57%	21.34%			41.78%	54.97%		23.23%
Lactococcus																											0.77%
Leclercia		21.11%	30.03%			0.18%				9.77%							27.94%		21.24%	17.22%					0.19%		
Leptotrichia						0.18%																1.96%		1.79%			
Megasphaera																							1.29%				
Methylobacterium														2.35%													
Neisseria	7.30%	1.68%	1.04%			0.20%							1.20%	4.26%									8.30%				0.17%
Paracoccus			1.27%							26.82%																	
Parvimonas																						4.24%					
Peptoniphilus						0.10%																					
Peptostreptococcus											1.51%																
Phreatobacter																								1.81%			
Phyllobacterium				2.08%																							
Porphyromonas	2.77%		1.54%			0.12%								1.44%								6.47%	5.66%		0.19%		
Prevotella	9.89%	8.38%	2.28%	0.65%	6.71%	2.56%					46.93%	3.30%	34.65%	20.25%			1.07%		2.75%	7.29%		17.03%	19.29%	6.13%	1.14%	1.39%	3.64%
Pseudomonas	3.05%				1.62%									1.18%													
Ralstonia	2.98%			1.50%	2.30%									3.31%									2.62%				
Rhizobium														8.17%													
Rothia					3.44%																	1.63%	1.11%				
Selenomonas																										1.36%	
Sphingomonas	1.40%			13.29%						2.65%					5.39%			5.70%			12.44%						
Stenotrophomonas				1.60%																							
Stomatobaculum														2.23%													0.16%
Streptococcus	3.64%	1.65%			19.31%	0.64%						1.09%	10.12%	1.73%	7.07%		1.25%	12.63%	4.95%	1.13%		12.18%	12.22%	8.94%	0.56%		0.17%
Tepidimonas					2.20%																						
Treponema																										9.37%	
Veillonella	1.38%				6.41%	0.30%					23.32%		9.60%	4.47%								5.12%	7.08%	6.62%	0.21%		0.47%
Xanthomonas																			1.66%								

Table S2. The percentages of bacteria species enriched in microbiota of gastric cancer patients

strain	gastritis									intestinal metaplasia							cancer										
Helicobacter pylori	0.01%	0.01%	0.02%	0.32%	10.94%	88.61%	94.61%	98.10%	98.53%	0.01%	0.03%	0.08%	6.18%	9.66%	67.54%	88.07%	0.00%	0.24%	0.03%	0.04%	0.04%	0.80%	0.93%	1.03%	4.73%	5.44%	16.72%
Clostridium colicanis	0.00%	0.02%	0.01%	0.00%	0.00%	0.00%	0.00%	0.00%	0.00%	0.00%	0.00%	0.00%	0.00%	0.00%	0.00%	0.00%	0.00%	0.00%	0.00%	0.00%	52.18%	3.67%	0.15%	0.23%	0.00%	24.10%	0.00%
Fusobacterium canifelinum	0.00%	0.00%	0.00%	0.00%	1.44%	0.02%	0.00%	0.00%	0.00%	0.00%	0.00%	0.00%	0.23%	0.00%	0.00%	0.00%	34.08%	1.59%	0.00%	0.00%	0.03%	0.00%	0.00%	0.37%	0.31%	0.02%	0.00%
Fusobacterium nucleatum	0.00%	0.16%	0.07%	1.38%	0.64%	0.09%	0.00%	0.00%	0.02%	0.00%	0.00%	0.04%	0.69%	0.37%	0.00%	0.01%	9.41%	5.25%	0.00%	4.32%	3.53%	0.00%	0.00%	0.44%	0.16%	0.22%	0.12%
Lactobacillus gasseri	0.00%	0.00%	0.00%	0.00%	0.04%	0.00%	0.00%	0.00%	0.00%	0.00%	0.00%	0.00%	0.00%	0.00%	0.00%	0.00%	0.02%	0.04%	6.03%	0.32%	6.95%	0.72%	0.00%	0.00%	2.07%	0.00%	0.00%
Lactobacillus reuteri	0.00%	0.00%	0.00%	0.00%	0.02%	0.00%	0.00%	0.00%	0.00%	0.00%	0.00%	0.00%	0.00%	0.00%	0.00%	0.00%	0.72%	0.01%	2.29%	0.25%	0.68%	0.07%	0.15%	0.00%	5.02%	0.00%	0.00%
Cc+Fn	0.00%	0.19%	0.08%	1.38%	0.64%	0.09%	0.00%	0.00%	0.02%	0.00%	0.00%	0.04%	0.69%	0.37%	0.00%	0.01%	9.41%	5.25%	0.00%	4.32%	55.71%	3.67%	0.15%	0.66%	0.16%	24.32%	0.12%
Cc+Fc+Fn	0.00%	0.19%	0.08%	1.38%	2.08%	0.11%	0.00%	0.00%	0.02%	0.00%	0.00%	0.04%	0.92%	0.37%	0.00%	0.01%	43.49%	6.84%	0.00%	4.32%	55.74%	3.67%	0.15%	1.03%	0.47%	24.34%	0.12%
Cc+Fc+Fn+Lg+Lr	0.00%	0.19%	0.08%	1.38%	2.14%	0.12%	0.00%	0.00%	0.02%	0.00%	0.00%	0.04%	0.92%	0.37%	0.00%	0.01%	44.23%	6.89%	8.32%	4.89%	63.38%	4.47%	0.30%	1.03%	7.56%	24.34%	0.12%

Abbreviation: *Cc, Clostridium colicanis; Fc, Fusobacteriumcanifelinum; Fn, Fusobacteriumnucleatum; Lg, Lactobacillus gasseri; Lr, Lactobacillus reuteri*
